# Anti-Biofilm Effect of Biodegradable Coatings Based on Hemibastadin Derivative in Marine Environment

**DOI:** 10.3390/ijms18071520

**Published:** 2017-07-13

**Authors:** Tiffany Le Norcy, Hendrik Niemann, Peter Proksch, Isabelle Linossier, Karine Vallée-Réhel, Claire Hellio, Fabienne Faÿ

**Affiliations:** 1 Laboratoire de Biotechnologie et Chimie Marines, Institut Universitaire Européen de la Mer, Université de Bretagne-Sud, 56100 Lorient, France; tiffany.le-norcy@univ-ubs.fr (T.L.N.); isabelle.linossier@univ-ubs.fr (I.L.); karine.rehel@univ-ubs.fr (K.V.-R.); 2 Institute of Pharmaceutical Biology and Biotechnology, Heinrich-Heine-University Düsseldorf, 40225 Düsseldorf, Germany; hendrik-niemann@web.de (H.N.); proksch@uni-duesseldorf.de (P.P.); 3 Biodimar, LEMAR UMR 6539, Institut Européen de la Mer, Université de Bretagne Occidentale, 29200 Brest, France; Claire.Hellio@univ-brest.fr

**Keywords:** antifouling, biodegradable coating, hemibastadin, sponges, marine bacteria, microalgae

## Abstract

Dibromohemibastadin-1 (DBHB) is an already known potent inhibitor of blue mussel phenoloxidase (which is a key enzyme involved in bioadhesion). Within this study, the potentiality of DBHB against microfouling has been investigated. The activity of DBHB was evaluated on key strains of bacteria and microalgae involved in marine biofilm formation and bioassays assessing impact on growth, adhesion and biofilm formation were used. To assess the efficiency of DBHB when included in a matrix, DBHB varnish was prepared and the anti-microfouling activity of coatings was assessed. Both in vitro and in situ immersions were carried out. Confocal Laser Scanning Microscopy (CLSM) was principally used to determine the biovolume and average thickness of biofilms developed on the coatings. Results showed an evident efficiency of DBHB as compound and varnish to reduce the biofilm development. The mode of action seems to be based principally on a perturbation of biofilm formation rather than on a biocidal activity in the tested conditions.

## 1. Introduction

Marine biofouling is a natural process, which is initiated by the adsorption of organic and mineral molecules, followed by the adhesion of microfouling (bacteria and microalgae principally) and macrofouling (macroalgae and invertebrates) subsequently. Biological fouling leads to detrimental economic and environmental impacts on marine surfaces (increase in roughness, corrosion, fuel consumption of ships, etc.). Several strategies have been developed to combat fouling based on self-polishing coatings (SPC) or controlled depletion paint (CDP). Most of them include the use of polymeric coatings (polyacrylic resins) blended with metals such as copper and/or toxic organic compounds [1]. Several modes of action were generally required for these coatings: hydration, hydrolytic degradation, erosion and biocides release. For almost 50 years, commercial antifouling (AF) paints used tributyltin (TBT), a broad-spectrum biocide formulated in copolymer paints with cuprous oxide. However, TBT was proven to be highly toxic for many aquatics organisms and marine ecosystems. Consequently, since 2008, the international maritime organization has banned the use of TBT in AF coatings. Today, coatings are formulated using copper (CuSCN, Cu_2_O or Cu metal) and organic molecules named booster biocides. Nevertheless, these solutions are not environmentally satisfactory. For example, copper concentration that exceeds 3.1 ppb (the US federal standard) affects various life stages of marine organisms including mussels, oysters, scallops, sea urchins and crustaceans [2]. SeaNine (4,5-dichloro-2-n-octyl-4-isothiazolin-3-one), a fungicide, has a high risk to dismantle the biotic community through rapid bioaccumulation in non-target benthic organisms [3]. Other biocidal organic pesticides and herbicides such as Diuron and Irgarol 1051 clearly damage marine organisms and the marine environment. Thus, many biocides are restricted or totally banned by several regulations including EU Biocidal Product Regulation (EU 528/2012). The development of molecules that inhibit or deter biofouling through non-biocidal mechanisms are searched actively.

Natural products from marine organisms can potentially be used as replacements for biocides commonly added in AF coatings. Indeed, many marine sessile organisms keep free from biofouling by producing bioactive metabolites with antifouling properties. AF substances have been extracted from a variety of marine organisms: microorganisms (marine bacteria, fungi, cyanobacteria), macroalgae, aquatic plants and marine invertebrates [4,5]. Different modes of actions are known: inhibitor of adhesive production/release, biofilms inhibitors, quorum sensing blockers, protein expression regulators, blockers of neurotransmission or surface modifiers [6,7].

Marine sponges are a rich source of bioactive metabolites [7,8,9,10] among which some have reported antifouling, antimicrobial, anti-biofilm and antibacterial activities [7]. For example, barretin and oroidin and their synthetic derivatives have been incorporated in paint formulations and have shown great potencies for biofouling inhibition in field studies [11,12]. One group of marine compounds described in the literature that display particular potential as AF agent are bromotyrosine-derived sponge metabolites [13]. Among them, bastadins, isolated from a marine sponge *lanthella basta*, are a promising source of AF compounds [14]. Some of them are able to inhibit barnacle adhesion and adhesives synthesis by mussel [15,16].

**Scheme 1:**
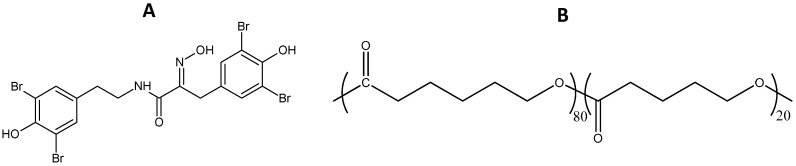
Chemical structure of: (**A**) Dibromohemibastadin-1 (DBHB); and (**B**) poly(ε-caprolactone-co-δ-valerolactone) (P(CL-VL)).

This study focuses on the use of a hemibastadin analog named dibromohemibastadin-1 (DBHB) (Scheme 1A) which was previously described as a non-toxic AF compound. It indeed inhibited the settlement of cyprid larvae of *Barnacle improvisus* and byssus formation of *Mytilus edulis* without toxicity on nauplii of *Artemia salina* [15,16,17]. However, no research has previously investigated the efficiency of new coatings based on DBHB as active agent.

This study reports the evaluation of a coating (called varnish) based on biodegradable polymer named poly(ε-caprolactone-co-δ-valerolactone) (P(CL-VL)) [18] incorporating hemibastadin derivative (Scheme 1B). P(CL-VL) composed of 80% caproic acid and 20% pentanoic acid is a good candidate to obtain CDP taking into account industrial constraints (film-forming properties, biodegradability, life-time, stability, biocide release, etc.) [19,20]. DBHB as compound and varnish has been evaluated by laboratory and field assays.

## 2. Results and Discussion

The results presented in this paper concern the evaluation of the biological activity of DBHB. The approach used: (i) the evaluation of the toxicity of DBHB against several bacterial and microalgal strains; (ii) the study of its activity against adhesion and biofilm formation; and (iii) the study of the activity of coatings incorporating DBHB against microfouling proliferation. For this last experiment, two conditions have been used: in a controlled medium (in vitro test) and in natural seawater (in vivo test).

### 2.1. Dibromohemibastadin (DBHB) Bioactivity

DBHB has been tested against single cultures of bacteria and diatoms. Three marine bacteria were chosen and four microalgal species. They were selected for their ability to form biofilm and to be involved in biofouling process [13,21,22].

#### 2.1.1. Anti-Bacterial Activity

**Figure 1:**
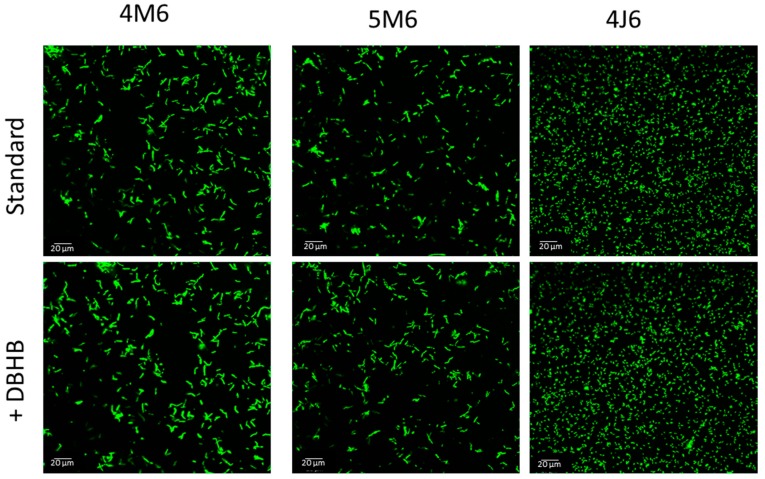
Impact of DBHB on the bacterial adhesion on glass surface at 20 °C without addition of DBHB (Standard) and containing DBHB at 16 μM (+DBHB). Adhesion of *Paracoccus* sp. (4M6), *Pseudolateromonas* sp. (5M6) and *Bacillus* sp. (4J6) was observed by DBHB and detected by staining the cells with Syto 9. DBHB: Dibromohemibastadin. Sale bar: 20 μm.

Results showed no effect of DBHB: the Minimal Inhibition Concentration (MIC) was higher than 80 μM. Concerning adhesion and biofilm formation, observations have been made by CLSM in the presence of 16 μM DBHB. The adhesion of bacteria on glass slide was not reduced by the presence of DBHB: no inhibition was observed (Figure 1).

**Figure 2:**
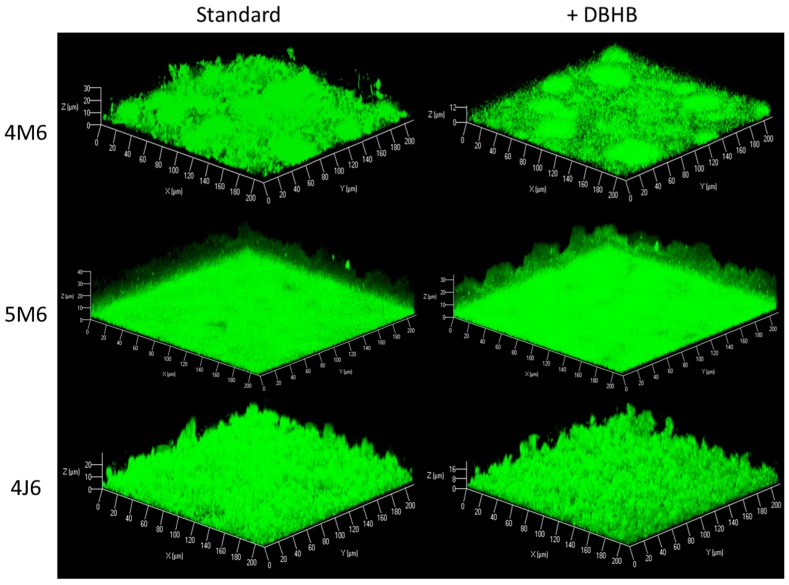
Impact of DBHB on the bacterial biofilm formation. CLSM observations of the bacterial biofilm grown on glass surface at 20 °C under a flow of medium without addition of DBHB (Standard) and containing DBHB at 16 μM (+DBHB). Biofilms of *Paracoccus* sp. (4M6), *Pseudoalteromonas* sp. (5M6) and *Bacillus* sp. (4J6) were detected by staining the cells with Syto 9.

However, as shown in Figure 2 and Table 1, a significant inhibition of biofilm formation (39.6%) was observed for *Paracoccus* sp. 4M6. Contrariwise, no impact of DBHB on *Pseudolateromonas* sp. 5M6 and *Bacillus* sp. 4J6 biofilm formation was observed. Moreover, the addition of DBHB induced no mortality in different biofilms as observed by Syto 9/Sytox red staining (results not shown): the mortality percent was lower than 1% for the three strains.

Natural products from marine organisms are sources of metabolites with biological activities, probably as a mean of protection from colonization [23]. They acted principally on the settlement of fouling organisms [7,23]. However, some mechanisms concern the bacterial biofilm (quorum sensing inhibitors, surface modifiers). In the case of sponges, some compounds prevent the cross talk “Quorum Sensing” during biofilm development [24,25,26]. For example, *Luffariella variella* extracts that contained specific sesterterpenoid were able to inhibit quorum sensing (QS) against both Gram-positive and Gram-negative bacteria [27]. A γ-lactone, extracted from the *Plakortis* cf. lita (plakofuranone), was able to inhibit QS [28]. Moreover, many natural brominated alkaloids have demonstrated antagonistic effects on QS [29]. Nevertheless, among compounds possessing AF and anti-biofilm properties, only few of them (terpenoids and pyrrole imidazoles) modulated biofilm formation without killing the bacteria or disrupting their growth [10].

In this study, DBHB has shown an interesting activity on biofilm formation on the marine bacterial strain *Paraccocus* sp. 4M6. Furthermore, this anti-biofilm activity was not connected to antibacterial effect at 16 μM. 4M6 is a Gram-negative bacteria, the single studied strain producing some AHL (C_4_, C_6_, C_8_, 3-oxo-C_12_) [21]. Hence, it would be hypothesized that DBHB could interact specifically with Acyl-homoserine lactone (AHL).

DBHB has four bromine groups. Several studies have already shown the anti-biofilm activity of brominated compounds [30], particularly, for molecules from bromotyrosine derivatives [13,31]. Previously, Andjouh and Blache [31] have shown that hemibastadins analogs were no lethal at 100 μM for to three marine bacteria whose *Paracoccus* sp. 4M6. Other derivative compounds such as oroidin, lanthelline or barettin from sponges have shown an antibacterial activity [8,13]. Nevertheless, these compounds had a more pronounced effect on the inhibition of marine bacterial growth and adhesion with MIC values below 10 μg/mL [13]. Others studies have shown that oroidin is able to interfere with bacterial attachment and showed moderate inhibitory activity against biofilm formation through a non-microbiocidal mechanism [8,32,33,34]. The anti-biofilm activity without absence of viability of brominated analogs was mainly obtained for a high level of bromination [35].

DBHB can inhibit biofilm formation of some bacterial strains (Gram-negative bacteria) without bacteriostatic and bacteriolytic activities. Given that Gram-negative bacteria are majority in marine environment [36] and that bacterial biofilm is considered as a cue for the further biofouling development, DBHB could be a potential interesting candidate as antifouling agent.

#### 2.1.2. Microalgae Activity

Results showed an effect on growth for *C. closterium* and *E. gayraliae* with a MIC values of 80 μM (50 μg/mL) after five days. *P. purpureum* and *H. coffeaeformis* remained unaffected at concentration of 80 μM. Results showed a low anti-microalgal activity and toxicity of DBHB against microalgae. For comparison, two similar brominated marine compounds (Ianthelline et Barettin) have shown an effect on growth (MIC from 0.1 μg/mL to >10 μg/mL) on four microalgae [13].

*C. closterium* is particularly interesting because it is known for its presence in marine biofilms. This species is deeply studied for its extracellular polymeric substances, its adhesion mechanisms and its relationships with bacteria [36,37,38]. It is able to form mixed biofilm with bacterial strains and a possible role of bacterial signal molecules AHL in the formation of its biofilm has been indicated [39,40,41].

**Figure 3:**
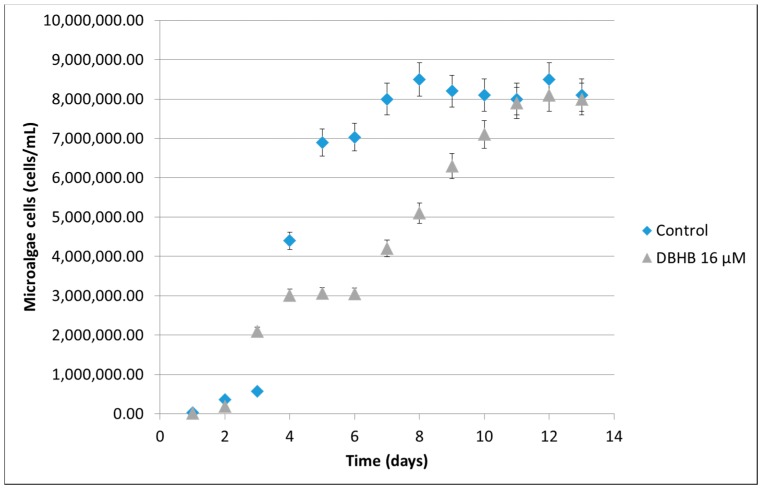
Influence of DBHB (16 μM) on bacterial growth. Data represent the mean ± standard deviation of replicates.

The growth kinetics of *C. closterium* in the presence of 16 μM of DBHB is shown in Figure 3. DBHB reduced the growth kinetics without any toxic effects. The stationary phase was reached after 14 days.

**Figure 4:**
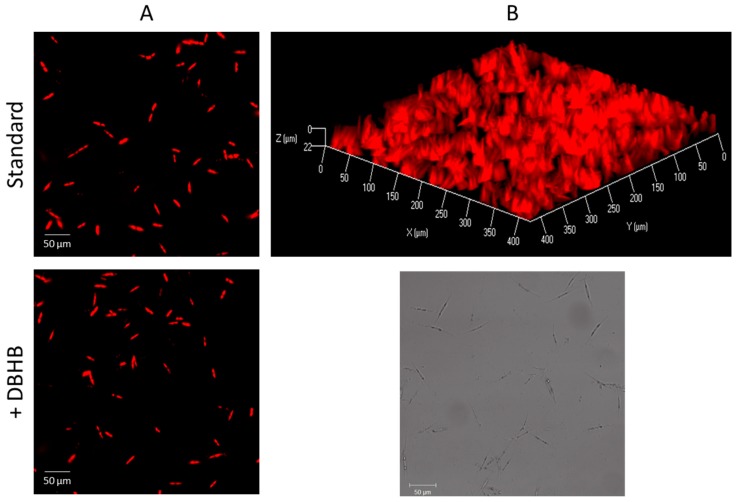
Effect of DBHB (16 μM) on: (**A**) *C*. *closterium* adhesion, scale bar: 50 μm (24 h); and (**B**) biofilm maturation (24 h). CLSM images represent adhered cells on the glass slide without the presence of DBHB (standard on the top of the panel) and in the presence of DBHB (at the bottom).

Microscopic observations of diatoms adhered after 24 h on glass slides in the presence of 16 μM are shown in Figure 4A. An adhesion decrease (14%) was quantified. Nevertheless, this result was not significant (*p* > 0.05). For a higher concentration (80 μM), the inhibition of adhesion was confirmed. After 24 h, the number of cells was 40% lower (*p* < 0.01) than the standard. Nevertheless, if the adhesion time is prolonged until 72 h, the cells number increased significantly (*p* < 0.05) from 2.0 × 10^4^ to 2.5 × 10^4^ cells/cm^2^). This result proved a lower growth.

After 24 h in presence of medium flow (150 μL/min) containing DBHB (16 μM), no diatom biofilm was obtained. A quenching of microalgae fluorescence was observed (Figure 4B). This effect indicated a loss of microalgae viability. This observation was confirmed for several concentrations (2, 8, 16 and 80 μM). In these experimental conditions, DBHB was toxic against *C*. *closterium*. This result can be explained by the presence of the flow involving mechanical stress. In dynamic conditions, *C. closterium* was sensitive at lower concentrations.

**Figure 5:**
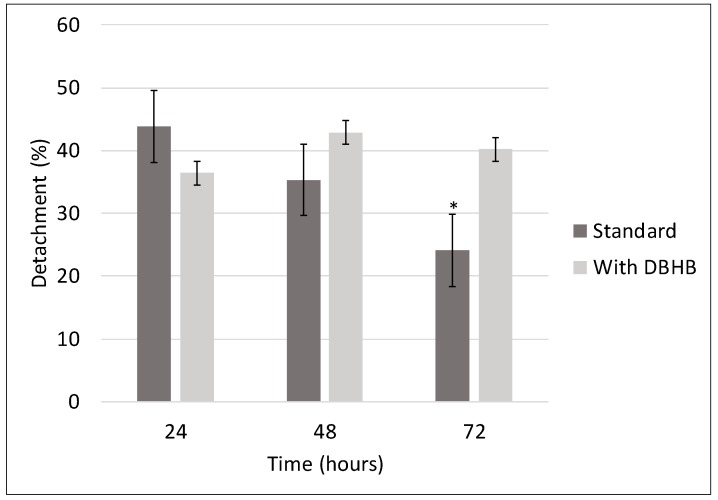
Impact of DBHB (16 μM) on detachment of *C. closterium* after 24, 48 and 72 adhesion. Data represent the mean ± standard deviation of replicates; * statistically significant difference at the 95% confidence level between standard (without DBHB) and treated samples.

The adhesion strength of diatoms is an important parameter in antifouling development [42]. Hence, the effect of DBHB on this parameter was evaluated (Figure 5). Diatoms detachment was quantified after 24, 48 and 72 h of adhesion by adding a flow (150 μL/min) corresponding to a shear stress of 62.5 Pa. For the standard, the detachment percent of diatoms from the glass surface decreased with adhesion time. It means that: an increase of adhesion strength was observed. On the contrary, in presence of DBHB, the detachment percent was independent of the adhesion time: values were not significantly different (*p* > 0.05) after 24, 48 or 72 h of adhesion. Nevertheless, after 72 h, the detachment percent was 1.7 times higher in the presence of DBHB than in standard conditions. These results indicated a significant decrease (*p* < 0.01) of diatoms attachment strength.

Hence, in term of anti-diatoms activity, DBHB acted differently from bacteria. A delayed growth was observed. In term of adhesion, few DBHB effects were observed in the range of tested concentrations. However, an interesting impact on the attachment strength was observed. In the presence of a flow, DBHB clearly impacted *C. closterium* viability: a toxic effect was observed. Moreover, previous studies have shown that bastadins derivative altered the intracellular Ca^2+^ levels inhibiting the attachment of the cypris larvae of barnacles and mussels phenoloxidase [15,17]. This mode of action, which concerned specific molecular targets could impact on microalgal adhesion. It was reported that Ca^2+^ might be involved in the mobility, attachment and growth of diatoms [43]. Moreover, a recent study has shown the effect of QS inhibitors on the growth and the diatom biofilm formation [40,41]. Thus, several mechanisms could explain the impact of DBHB on diatoms biofilm formation.

DBHB can perturb bacterial and diatoms biofilm. Both these types of microorganisms represent the major component of microfouling which constitutes an important factor for the formation of biofouling. Thus, the potentiality of the hemibastadin derivative as AF agent is interesting to further study in coatings.

### 2.2. In Vitro Activity of Coatings

**Figure 6:**
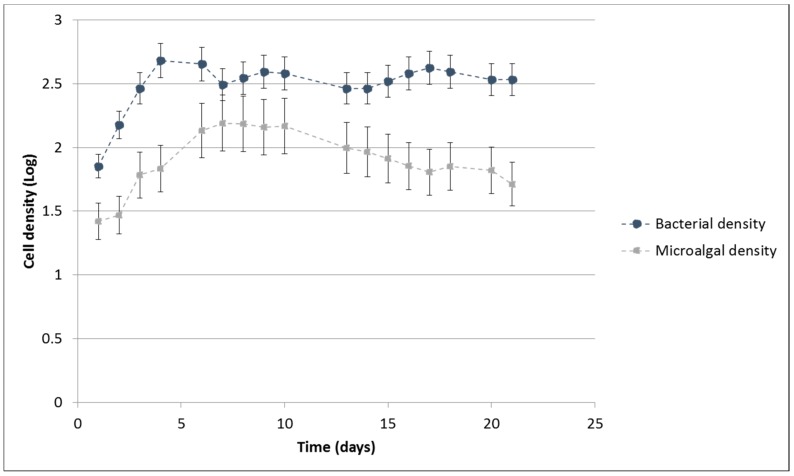
Planktonic cell density for the co-culture of bacteria (units: DO_600nm_) and microalgae (units: arbitrary units of fluorescence, λ_excitation_ = 633 nm, λ_emission_ = 638–720 nm) in photobioreactor. Data represent the mean ± standard deviation of replicates.

After dissolution in xylene at a concentration of 0.1 mg/mL, DBHB was added to a poly(ε-caprolactone-co-δ-valerolactone) solution. After mixing and application on a polycarbonate surface, a homogeneous film was obtained. Efficiency of coatings was observed following simultaneous exposure to a multi-species culture in photo-bioreactor. The system was inoculated with three bacterial strains (*Paracoccus* sp. 4M6, *Bacillus* sp. 4J6 and *Pseudoalteromonas* sp. 5M6) and one diatom strain (*C. closterium*). Bacteria and diatoms are very close in fouling biofilms [44], and they might support each other by exchange of chemical cues [45]. The mixed biofilm, constituted of bacteria and diatoms in symbiotic relation, can establish a resistant consortium against AF agents. Moreover, the efficiency of DBHB against *Paracoccus* sp. 4M6 and *C. closterium* should decrease in the presence of other bacteria. Hence, marine bacteria and diatom were co-cultivated and biofilm formation was observed during 21 days (Figure 6). During experimentation time, the bacterial and diatoms cells number increased to reach a stationary phase after six days. The cells densities were 2.9 × 10^6^ and 7.7 × 10^5^ cells/mL, respectively. No toxic effect of coatings against bacteria and diatoms was observed in the condition of the experiment. Moreover, antibacterial and anti-microalgal activities were assayed by the diffusion method, which consists of measuring the halo of inhibition around a piece of coating. No inhibition was observed for bacterial and diatom strains. DBHB coating was not toxic against studied strains in these conditions. Two reasons can explain this fact:

The amount of DBHB released is lower than MIC. The quantification of DBHB concentration in surrounding medium was realized by LC-MS-MS (limit of quantification: 20 μM). No presence of DBHB was observed during all experiment, which implied a potential amount in the medium lesser than MIC.

The polymeric matrix used is biodegradable and no toxic [19].

**Figure 7:**
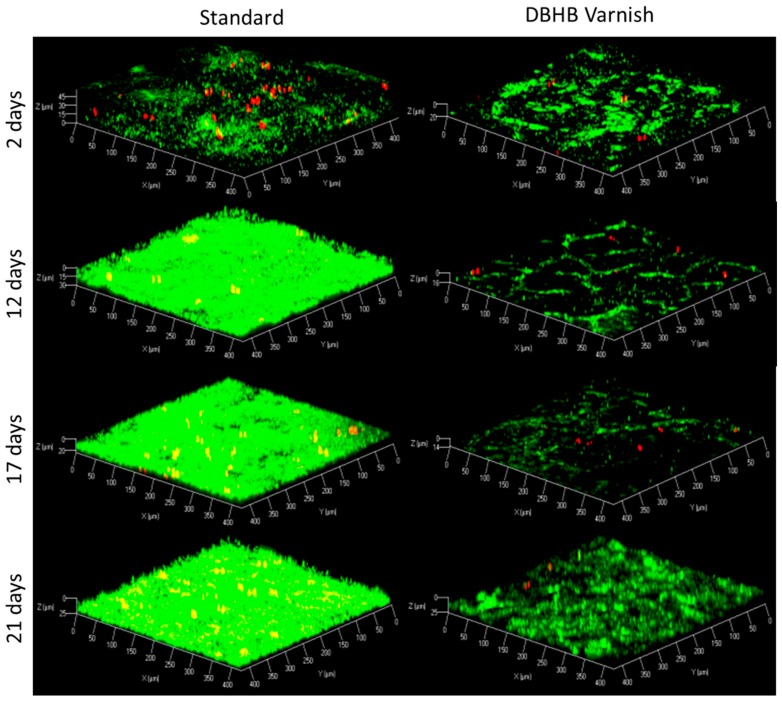
CLSM photography of microfouling developed on varnishes containing DBHB (DBHB varnish) and without DBHB (standard) during in vitro immersion. The mixed cultures were realized in PBR. Color allocation: red, autofluorescence of chlorophyll; green (Syto 9), bacteria; yellow: overlay of microalgae and bacteria

**Figure 8:**
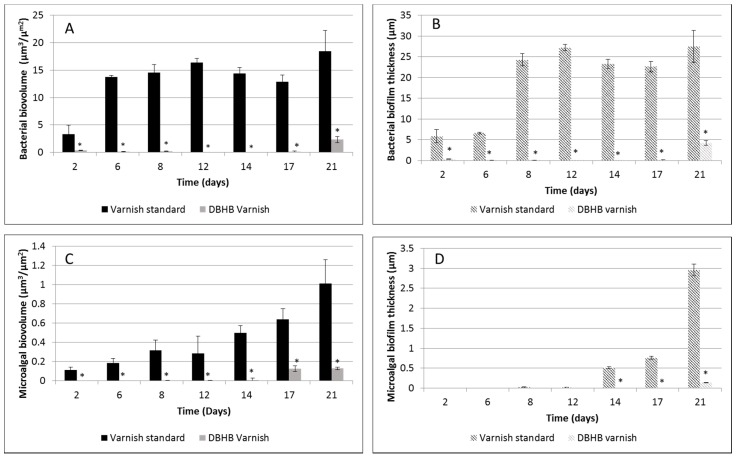
Biofilm quantification on varnishes during in vitro immersion. Bacterial and microalgal biovolumes (**A**,**C**) and biofilm thicknesses (**B**,**D**) were determining during 21 days after COMSTAT analysis of CLSM observations. Data represent the mean ± standard deviation of replicates; * statistically significant difference at the 95% confidence level between standard (varnish without DBHB) and varnish containing DBHB.

DBHB varnish was compared with standard (without DBHB). The mixed biofilms formed at the surface of coatings were observed by CLSM (Figure 7) and quantified (Figure 8). Bacterial and diatoms biomasses were significantly (*p* < 0.05) lower on DBHB-varnish than on standard. DBHB varnish exhibited exceptionally low biomasses. At 12 days, reductions of 99% and 71% of bacterial and diatoms species were observed, respectively. In addition, the thickness of the mixed biofilm was significantly (*p* < 0.01) reduced on DBHB-varnish versus on the standard. For example, after 21 days, average bacterial biofilm thickness was significantly (*p* < 0.01) reduced from 27 to 4 μm for standard and DBHB varnish respectively. In terms of diatoms, average biofilm thicknesses were lower by an order of magnitude of 10 for DBHB varnish.

The evaluation of the development of mixed biofilm on marine coatings is poorly studied in the literature: only two papers to our knowledge [46,47]. Coatings studied in these papers are amphiphilic fouling release (FR) coatings. Their foul release behavior is attributed to their physical properties and not the presence of anti-biofilm molecules. However, the inhibition percent’s were in the same magnitude order: 80% average.

**Figure 9:**
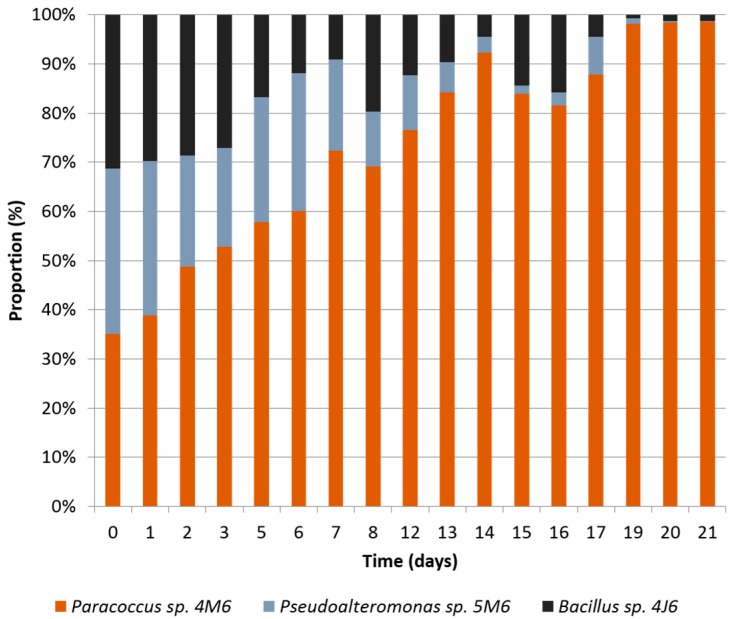
Proportion of planktonic bacterial strains in Photobioreactor (PBR) during experiment. The determination was realized by enumeration on agar plate. Data represent the mean of replicates.

In vitro test confirmed the anti-biofilm activity of DBHB varnish by acting on bacteria and diatoms. Antibacterial assays presented above showed that only *Paracoccus* sp. 4M6 was affected by the presence of DBHB. In the presence of the three bacteria, 4M6, 5M6 and 4J6, the effect of DBHB on bacterial biofilm was conserved. This result can be explained by relations, which are established between various bacteria (competition, commensalism, parasitism and other microbiological process). The proportion of the three bacteria in the culture medium during experiment (Figure 9) showed a regular decrease of 4J6 and 5M6 cells number in favor to 4M6. Results showed that 4M6 inhibited the growth of 4J6 and 5M6. The mixed biofilms observed on coatings were essentially composed of 4M6 and *C. closterium*.

### 2.3. In Vivo Activity of Varnishes

**Figure 10:**
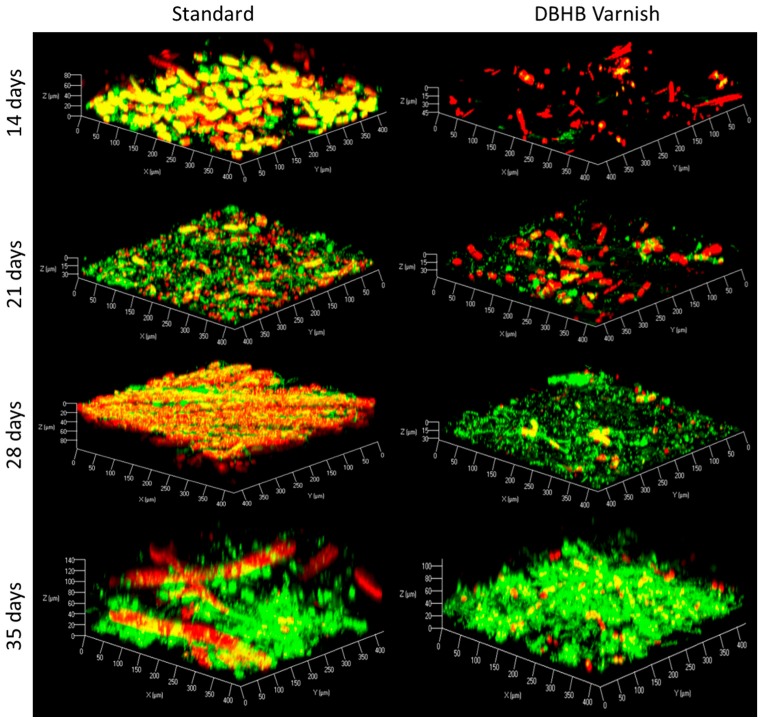
CLSM photography of microfouling developed on varnishes containing DBHB (DBHB varnish) and without DBHB (standard) during in vitro immersion (Kernevel Harbour). Color allocation: red, autofluorescence of chlorophyll; green (Syto 9), bacteria; yellow: overlay of microalgae and bacteria.

**Figure 11:**
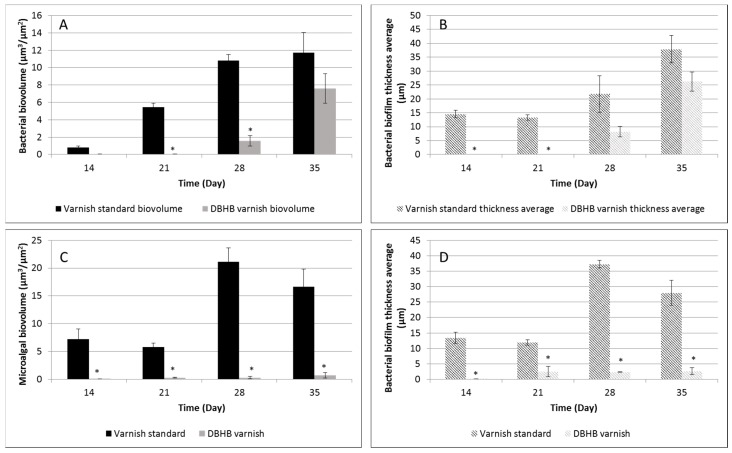
Biofilm quantification on coatings during in vivo immersion. Bacterial and microalgal biovolumes (**A**,**C**) and biofilm thicknesses (**B**,**D**) were determining during 35 days after COMSTAT analysis of CLSM observations. Data represent the mean ± standard deviation of replicates; * statistically significant difference at the 95% confidence level between standard (varnish without DBHB) and varnish containing DBHB.

To evaluate the anti-microfouling activity in actual conditions, coatings were immersed on a raft in the Atlantic Ocean. Microfouling (bacteria and microalgae) was observed at 14, 21, 28 and 35 days. Results of anti-microfouling activity during the 35 days of immersion are shown in Figure 10 and Figure 11. A biofilm composed of a diversity of microorganisms was rapidly developed on the standard. Micrographs and bioadhesion analysis revealed that DBHB-varnish was more efficient than standard against microfouling. Nevertheless, differences were observed between bacteria and microalgae in term of activity. Bacterial and diatoms biomass developed on DBHB-varnish was significantly (*p* < 0.05) lower than standard during the 28 first days. After 35 days in natural seawater, both coatings were totally covered by bacteria and the biovolume quantified on DBHB varnish was lower but not significantly different from standard. This result can be explained by the heterogeneity of bacterial biovolume on the coating surface causing large standard deviations. Contrariwise, the development of microalgae on DBHB varnish remained significantly weaker than on standard: the microalgae biovolumes were 16.64 ± 3.2 μm^3^/μm^2^ and 0.71 ± 0.5 μm^3^/μm^2^ respectively and the average thicknesses were 27.99 ± 4.05 μm and 2.61 ± 1.18 μm, respectively. The inhibition rates were estimated at 78% and 91% for microalgae biovolume and average thickness.

Only a few studies have focused on the use of CLSM to study biofilm development on antifouling paints [20,48,49]. CLSM make it possible to represent the 3D-architecture of the sample and to extract quantitative structural parameters such as the biofilm biovolume and thickness. However, the non-control of the physico-chemical properties of seawater (location, seasonality) [50,51] and the variability of coatings (varnish versus paint, AF versus FR) does not allow a formal comparison between these studies. Nevertheless, it is possible to compare the inhibition rate of microfouling. In Loriot et al. [20], antifouling paints based on P(CL-VL) and commercial CDP was studied and compared to standard without biocide. The rate of inhibition was 95% on average for diatom biomass and maximum thickness after six weeks.

### 2.4. Potentiality of DBHB Coating

The evaluation of DBHB coating against microfouling has been tested by using in vitro and in situ assays. The biovolume data are compiled in Table 2.

Results obtained in vitro and in situ methods corroborated. DBHB coating inhibited significantly (*p* < 0.01) the microfouling adhesion. The bacterial inhibition percent quantified on DBHB varnish comparatively to standard was not significantly different (*p* < 0.05), whereas a significant difference of 10% was observed in term of microalgal adhesion (*p* < 0.01). This difference could be explained by the nature of microalgae. In natural conditions, several species of microalgae are presented, whereas in controlled conditions, only diatoms were evaluated. Nevertheless, both methods (in vitro and in situ) showed an effective efficiency of DBHB coating against microfouling.

It was important to highlight that DBHB concentration (0.02%) in coating was extremely low. Indeed, the typical concentration of common biocides (booster biocide and copper derivative) in commercial coatings is 20–40% average. Only few studies deal with the use of natural compound in coatings. However, these studies indicate higher concentrations than in our study. For example, butenolide, a compound derived from marine bacteria *Streptomyces*, showed an interesting antifouling activity in coating by incorporating from 10 to 15% of compound [52]. Tannins from chestnut, mimosa and quebracho trees were incorporated at a rate of 16% [53]. However, in the case of extracts from sponges, some studies indicated lower concentrations in coatings (1%) [54]. Sjögren et al. [12] included barettin and 8,9-dihydrobarettin in paints at the concentrations of 0.1%. Results confirmed the efficiency of coatings based on sponge extracts and their analogs at low concentration.

The potential mechanism of DBHB concerns an anti-biofilm effect rather than a biocidal effect. Results showed no bactericidal effect and only a weak microalgicidal effect under certain conditions. This mode of action was particularly sought as part of Product Biocide Regulation. Moreover, the use of biodegradable polymer did not inhibit activity of DBHB and was respectful of the environment. Hence, coatings could be used for all surfaces (fishing nets, oyster cages) and structures immersed in marine environment without toxicity. However, a clear description of mode of action, biological targets and environmental fate of new products is required by the regulation. Hence, the mode of action of DBHB against bacteria and diatoms should be discussed in a further works, particularly its potential anti-QS effect. The eco-toxicological effects of DBHB and its coating will be evaluated. Moreover, the incorporation of DBHB in paint’s formulation should make it possible to increase the duration effectiveness of the coating to one year.

## 3. Materials and Methods

### 3.1. Microorganism’s Strains and Growth

All the selected strains are involved in surface colonization [55].

Three marine bacteria, *Paracoccus* sp. 4M6 (Gram-negative), *Pseudoalteromonas* sp. 5M6 (Gram-negative) and *Bacillus* sp. 4J6 (Gram-positive) were selected in this study. They were isolated from the Gulf of Morbihan, France [19]. Strains were cultivated at 20 °C using Zobell media (Artificial SeaWater (ASW) 30 g/L, Tryptone 4 g/L, Yeast Extract 1 g/L). Bacterial cultures were incubated at 10^6^ CFU/mL during 48 h under agitation.

Four axenic microalgal strains, *Cylindrotheca closterium* AC170, *Exanthemachrysis gayraliae* AC 15, *Halamphora coffeaeformis* AC 713 and *Porphyridium purpureum* AC 122 were obtained from Algobank (Caen, France). The axenization was realized by a treatment with a mixture of three antibiotics: chloramphenicol (100 μg/mL), penicillin (1000 μg/mL) and streptomycin (500 μg/mL) during 24 h. The four strains were grown in sterile ASW medium with Guillard’s F/2 at 20 °C for 5 days under controlled illumination (250·μmol·photons·m^−2^·s^−1^) with a 12:12 h light–dark cycle. This procedure favored the cellular growth [56].

### 3.2. Anti-Bacterial Activities in Microplate

DBHB was evaluated at 0.01, 1, 5, 10, 20 and 50 μg/mL (0.016 to 80 μM) in twelve replicates in 96-well microplate (polypropylene, Fisher-Scientific, Waltham, MA, USA) [57]. Methanol was used as a carrier solvent to add DBHB in the microplate. Then an evaporation of solvent was performed (2 h). Then, 150 μL bacterial cultures of 48 h at 10^6^ CFU/mL were added in the wells containing the DBHB. Controls with methanol, without compound and the medium without cells were added. Microplates were incubated at 20 °C during 48 h. After incubation, the optical density at 600 nm was measured (TECAN, Magellan, Männedorf, Swiss) [13]. The minimum inhibitory concentration (MIC) was defined as the minimum concentration resulting in change in optical density increase after incubation [58]. Experiment was realized three times.

### 3.3. Anti-Microalgal Activities in Microplate

DBHB was evaluated at the same concentrations as mentioned above in 96-well microplate (black with transparent base, Fisher-Scientific) [57]. One hundred microliters microalgal cultures of 2 weeks at 10^3^ CFU/mL were added in the microplate containing the DBHB and microplates were incubated at 20 °C during 5 days under controlled illumination (250·μmol·photons·m^−2^·s^−1^) with a 12:12 h light–dark cycle. The same controls as above were used. Then, the fluorescence was measured (λ_excitation_ = 633 nm, λ_emission_ = 638–720 nm) to quantify the total cells density in each wells. MIC were defined as the lowest concentration that produced a significant reduction in growth. Experiment was realized three times.

### 3.4. Anti-Adhesion Assay

Experiments were realized in flow cell [59] for the three bacterial strains and one diatom strain *C. closterium*. For diatoms a specific protocol was developed. Cells were adhered on glass surface in three-channel flow cells. The system was assembled by sticking a microscope coverslip slide (24 × 50 mm). After a sterilization of the system by a flow of bleach (1.5%) during 24 h at 430 μL/min, a flow of ASW (30 g/L) was activated to clean and prepare the system for the bacterial or diatoms injection at 150 μL/min during 2 h.

Channels were inoculated with 250 μL of overnight bacterial cultures diluted in ASW medium (10^6^ CFU/mL) or 250 μL of 2 weeks diatoms cultures in F/2 medium (10^5^ CFU/mL). The flow cell was placed upside down to facilitate the cells adhesion on the glass. For bacterial and diatoms adhesion, the incubation temperature was 20 °C. For diatoms adhesion, a 12:12 h light–dark cycle was maintained. The incubation time was to 2 h for bacteria strains and 24 h for diatoms.

To quantify the anti-adhesion activity, DBHB was added in the flow cell at 16 μM in the bacterial or diatoms inoculum immediately prior to the injection. A condition without addition of DBHB was used as control. After 2 and 24 h of adhesion for bacteria and diatoms respectively, the flow at 120 μL/min was activated during 30 m to remove free bacteria and diatoms.

Cell adhesion was observed with confocal laser scanning microscopy (Zeiss, LSM 710, Oberkochen, Germany) by using a 40× oil immersion objective for bacteria and 20× air objective for diatoms. Adhered bacteria were observed with Syto9 nucleic acid stain (5 μM, λ_excitation_ = 488 nm, λ_emission_ = 498–540 nm). Adhered diatoms were observed by their fluorescence (λ_excitation_ = 633 nm, λ_emission_ = 638–720 nm). The overlap percentage was determined with a JAVA program (Université de Bretagne-sud, Lorient, France). Experiment was realized three times.

### 3.5. Diatoms Detachment Assay

After the adhesion stage, a protocol was developed to evaluate diatoms detachment. The flow was activated at 150 μL/min (shear stress = 62.5 Pa) during 30 min. Then, the cells number adhered on the surface were counted again. By comparing of cells number prior and after flow activation, it was possible to calculate the cellular detachment percentage. Experiment was realized three times.

### 3.6. Antibiofilm Assay

To observe the maturation of biofilm, after the adhesion step without the presence of DBHB, a flow of medium growth (Zobell and F/2 medium for marine bacteria and diatoms) at 1.5 ppm (120 μL/min) was activated for 48 h. The impact of DBHB on biofilm formation was evaluated by adding DBHB in the growth medium flow, and by comparing with the control condition without DBHB. The resulting biofilm was observed by CLSM as described above. The biovolume and the average thickness were determined with the COMSTAT program [60]. Experiment was realized three times.

The viability of bacterial biofilm was measured by syto9/sytox red nucleic acid stain (λ_excitation_ = 488 and 633 nm and λ_emission_ = 498–540 and 645–695 nm respectively). The viability percent was determined from biovolume of living and dead cells.

### 3.7. Coatings Preparation

A biodegradable polymer, the poly(ε-caprolactone-co-δ-valerolactone) was synthesized following [18] and characterized by ^1^H and ^13^C NMR in CDCl_3_ (500 MHz, Bruker Biopin SAS, Billerica, MA, USA) and Gel Permeation Chromatography with Polystyrene standard (Easical PS-2) in THF. It was composed of 80 mol % of caproic acid units and 20 mol % of valeric units. The molecular weight was 20,000 g·mol^−1^.

Varnishes were formulated from both solutions. The first contained 50% (*w*/*w*) of P(CL-VL) in xylene. Then DBHB in solution in methanol was added to obtain a 0.1 mg/mL final concentration. The solution was mixed by using a vortex (1 min) and ultrasounds (15 m, three times) at 20 °C. A layer of coating was deposited with an automatic film applicator (ASTM D823 Sheen instrument, Saint Denis, France) on a polycarbonate support. The coatings were dried at 20 °C during 24 h. After drying, DBHB represents 0.02% of dry extract. A varnish composed of P(CL-VL) without DBHB was used as a negative control. Obtained coatings was smooth and homogeneous.

### 3.8. Anti-Bacterial and Anti-Microalgal Activity of Coatings

Antibacterial and anti-microalgal activities were assayed by the diffusion method. It consisted in measuring the halo of inhibition around coatings. For this purpose, suspensions of bacteria or diatoms (100 μL) were inoculated onto Marine Broth (Fisher-Scientific) or ASW supplemented on F/2 agar present in Petri dishes (90 mm). Piece of coatings (diameter of 2 cm) were placed on the surface of agar plate. Varnish without DBHB was used as negative control. After an incubation period (48 h and 5 days at 20 °C for bacteria and diatoms respectively), the inhibition zone was measured. These experiments were repeated 6 times for each varnish.

### 3.9. Mixed Biofilm Test Conditions

A method in photo-bioreactor (PBR) was carried out. Prior to inoculation, coupons were placed in PBR (Eppendorf: Bioflow, CelliGen 115) containing 4 L of medium containing ASW (30 g/L) supplemented with F/2 (2%) and casein peptone (1 g/L). Microalgae pure culture was inoculated in the PBR at 1 × 10^3^ cells/mL (0.8 arbitrary units of fluorescence). Then 2 days after, 1 × 10^6^ cells/mL (0.25 optical density) of bacterial cultures (composed of the three bacteria) was added. The biofilm development on coupons (2 × 5 cm) that were deposed on the bottom of the PBR before inoculation was followed during 21 days. The growth parameters were maintained at 20 °C, pH 7.5, under controlled illumination 250 μmol photon m^−2^·s^−1^, an oxygenation of 50% and a constant agitation of 150 rpm. Every minute, 0.5 mL of sterile culture medium was fed to the photobioreactor and 0.5 mL was withdrawn by a peristaltic pump to avoid the lack of nutriment. The biofilm development was followed by collecting three coupons and 1 mL of the culture medium (planktonic microorganisms) was collected at each sample time (2, 6, 8, 12, 14, 17, 21 days). After collecting, each coupon was observed by CLSM as described above. Planktonic microorganisms density was evaluated by optical density (600 nm) and fluorescence (λ_excitation_ = 633 nm, λ_emission_ = 638–720 nm) measurements for bacteria and diatoms respectively. Moreover, the proportion of each bacteria strains was determined by counting on Marine Broth agar (Fisher Scientific). Experiment was realized three times.

### 3.10. Natural Seawater Exposition

Triplicate varnishes coupons (2 × 5 cm) were exposed in natural seawater, at a depth of 50 cm (Atlantic Ocean, W 47°42′20.779″ N 3°23′0.679″ W, Larmor Plage, France). The study began in April 2016. The seawater characteristics were: pH = 7.9–8.7, Oxygen = 6.8–12 mg/L, Temperature = 17.1–20.2 °C, Conductivity = 32.0–49.4 mS/cm.

Coupons were analyzed over 35 days and observed by CLSM microscopy, as described above [48]. For each sample times, three coupons were analyzed. Adhered bacteria and microalgae were observed at 14, 21, 28 and 35 days. Biovolumes and average thicknesses values were determined with COMSTAT program to compare both varnishes and to calculate an inhibition percentage.

### 3.11. Statistical Analysis of Data

Statistical analyses of the data obtained for all experiments were carried out using the one-factor analysis variance (ANOVA). *p* values of < 0.01 were considered as significant. Values are means ± standard deviation.

## Figures and Tables

**Table 1:** Percentage of inhibition of bacterial biofilms in the presence of DBHB (16 μM).

4M6	5M6	4J6
39.6%	0.11%	0.18%

**Table 2:** Comparison of bacterial and microalgae biovolumes developed on coatings during in situ and in vitro experiments.

Microorganisms	Coatings	In Situ ^1^	In Vitro ^2^
Bacterial biovolume (μm^3^/μm^2^)	Varnish	10.8 ± 0.7	18.4 ± 3.8
	DBHB varnish	1.6 ± 0.8	2.3 ± 0.5
	Inhibition (%)	85	88
Microalgal biovolume (μm^3^/μm^2^)	Varnish	21.1 ± 2.5	1.1 ± 0.2
	DBHB varnish	0.3 ± 0.2	0.13 ± 0.03
	Inhibition (%)	99	88

^1^ Experiment realized during 28 days. ^2^ Experiment realized during 21 days.

## Abbreviations

AF	Antifouling
AHL	Acyl homoserine lactone
ASW	Artificial Seawater
CDP	Controled Depletion Paint
CLSM	Confocal Laser Scanning Microscopy
DBHB	Dibromohemibastadin-1
FR	Foulinf Release
MIC	Minimal inhibition Concentration
PBR	Photo-bioreactor
P(CL-VL)	Poly(ε-caprolactone-co-δ-valerolactone)
QS	Quorum Sensing
SPC	Self Polishing Coatings
TBT	Tributyltin

## References

[1] Soroldoni S., Abreu F., Castro I.B., Duarte A., Lopes Leaes Pinho G. (2017). Are antifouling paint particles a continuous source of toxic chemicals to the marine environment?. J. Hazard. Mater..

[2] Carson R.T., Damon M., Johnson L.T., Gonzalez J.A. (2009). Conceptual issues in designing a policy to phase out metal-based antifouling paints on recreational boats in San Diego Bay. J. Environ. Manag..

[3] Cima F., Bragadin M., Ballarin L. (2008). Toxic effects of new antifouling compounds on tunicate haemocytes: I. Sea-Nine 211™ and chlorothanlonil. Aquat. Toxicol..

[4] Qian P.Y., Li Z., Xu Y., Li Y., Fusetani N. (2015). Mini-review: Marine natural products and their synthetic analogs as antifouling compounds: 2009–2014. Biofouling.

[5] Qian P.Y., Xu Y., Fusetani N. (2010). Natural products as antifouling compounds: Recent progress and future perspectives. Biofouling.

[6] Qian P.Y., Chen L., Xu Y. (2013). Mini-review: Molecular mechanisms of antifouling compunds. Biofouling.

[7] Satheesh S., Ba-akdah M.A., Al-Sofyani A.A. (2016). Natural antifouling compound production by microbes associated with marine macroorganisms—A review. Electron. J. Biotechnol..

[8] Kelly S.R., Jensen P.R., Henkel T.P., Fenical W., Pawlik J.R. (2003). Effects of Caribbean sponge extracts on bacterial attachment. Aquat. Microb. Ecol..

[9] Nogata Y., Kitano Y., Fusetani N., Clare A.S. (2006). Isocyano Compounds as Non-toxic antifoulants. Antifouling Compounds.

[10] Stowe S.D., Richards J.J., Tucker A.T., Thompson R., Melander C., Cavanagh J. (2011). Anti-biofilm compounds derived from marine sponges. Mar. Drugs.

[11] Melander C., Moeller P.D.R., Ballard E., Richards J.J., Huigens R.W., Cavanagh J. (2009). Evaluation of dihydrooroidin as an antifouling additive in marine paint. Int. Biodeterior. Biodegrad..

[12] Sjögren M., Dahlström M., Göransson U., Jonsson P.R., Bohlin L. (2004). Recruitement in the field of balanus improvises and mytilus edulis in response to the antifouling cyclopeptides barettin and 8,9-dihydrobarettin from the marine sponge geodia barrette. Biofouling.

[13] Hanssen K., Cervin G., Trepos R., Petitbois J., Haug T., Hansen E., Andersen J.H., Pavia H., Hellio C., Svenson J. (2014). The bromotyrosine derivative lanthelline isolated from the artic marine sponge stryphnus fortis inhibits marine micro- and macrobiofouling. Mar. Biotechnol..

[14] Fusetani N. (2004). Biofouling and antifouling. Nat. Prod. Rep..

[15] Bayer M., Hellio C., Maréchal J.P., Walter F., Lin W., Weber H., Proksch P. (2011). Antifouling bastadin congeners target mussel phenoloxidase and complex copper(II) ions. Mar. Biotechnol..

[16] Niemann H., Hagenow J., Chung M.Y., Hellio C., Weber H., Proksch P. (2015). SAR of sponge-inspired hemibastadin congeners inhibiting blue mussel phenoloxidase. Mar. Drugs.

[17] Ortlepp S., Sjögren M., Dahlström M., Weber H., Ebel R., Edrada R., Thoms C., Schupp P., Bohlin L., Proksch P. (2007). Antifouling activity of bromotyrosine-derived sponge metabolites and synthetic analogues. Mar. Biotechnol..

[18] Faÿ F., Renard E., Langlois V., Linossier I., Vallée-Rehel K. (2007). Development of poly(ε-caprolactone-co-l-lactide) and poly(ε-caprolactone-co-δ-valerolactone) as new degradable binder used for antifouling paint. Eur. Polym. Sci..

[19] Carteau D., Vallée-Réhel K., Linossier I., Quiniou F., Davy R., Compère C., Delbury M., Faÿ F. (2014). Development of environmentally friendly antifouling paints using biodegradable polymer and lower toxic substances. Prog. Org. Coat..

[20] Loriot M., Linossier I., Vallée-Réhel K., Faÿ F. (2017). Influence of biodegradable polymer properties on antifouling paints activity. Polymers.

[21] Grasland B., Mitalane J., Briandet R., Quemener E., Meylheuc T., Linossier I., Vallée-Réhel K., Haras D. (2003). Bacterial biofilm in seawater: Cell surface properties of early-attached marine bacteria. Biofouling.

[22] Dheilly A., Soum-Soutéra E., Klein G.L., Bazire A., Compère C., Haras D., Dufour A. (2010). Antibiofilm activity of marine bacterium *Pseudoalteromonas* sp. strain 3J6. Appl. Environ. Microbiol..

[23] Almeida J.R., Vasconcelos V. (2015). Natural antifouling compounds: Effectiveness in preventing invertebrate settlement and adhesion. Biotechnol. Adv..

[24] Müller W.E.G., Wang X., Proksch P., Perry C.C., Osinga R., Gardères J., Schröder H. (2013). Principles of biofouling protection in marine sponges: A model for the design of novel biomimetic and bio-inspired coatings in the marine environment?. Mar. Biotechnol..

[25] Quintana J., Brango-Vanegas J., Costa G.M., Castellanos L., Arévalo C., Duque C. (2015). Marine organisms as source of extracts to disrupt bacterial communication: Bioguided isolation and identification of quorum sensing inhibitors from Ircinia felix. Revis. Bras. Farmacogn..

[26] Saurav K., Bar-Shalom R., Haber M., Burgsdorf I., Oliviero G., Costantino V., Morgenstern D., Steindler L. (2016). In search of alternative antibiotic drugs: Quorum-quenching activity in sponges and their bacterial isolates. Front. Microbiol..

[27] Skindersoe M., Etting-Epstein P., Rasmussen T., Bjarnsholt T., de Nys R., Givskov M. (2008). Quorum sensing antagonism from marine organisms. Mar. Biotechnol..

[28] Costantino V., Della Sala G., Saurav K., Teta R., Bar-Shalom R., Mangoni A., Steindler L. (2017). Plakofuranolactone as a quorum quenching agent from the Indonesian sponge plakortis cf. lita. Mar. Drugs.

[29] Peters L., Konig G.M., Wright A.D., Pukall R., Stackebrandt E., Eberl L., Riedel K. (2003). Secondary metabolites of Flustra foliacea and their influence on bacteria. Appl. Environ. Microbiol..

[30] Sacristan-Soriano O., Banaigs B., Becerro M. (2012). Temporal trends in the secondary metabolite production of the sponge Aplysina aerophoba. Mar. Drugs.

[31] Andjouh S., Blache Y. (2015). Click-based synthesis of bromotyrosine alkaloid analogs as potential anti-biofilm leads for SAR studies. Bioorg. Med. Chem. Lett..

[32] Ballard T.E., Richards J.J., Wolfe A.L., Melander C. (2008). Synthesis and antibiofilm activity of a second-generation reverse-amide oroidin library: A structure-activity relationship study. Chem. Eur. J..

[33] Richard J.J., Ballard T.E., Huigens R.W., Melander C. (2008). Synthesis and screening of an oroidin library against Pseudomonas aeruginosa biofilms. Chembiochem.

[34] Olofson A., Yakushijin K., Horne D.A. (1998). Synthesis of marine sponge alkaloids oroidin, clathrodin, and dispacamides. Preparation and transformation of 2-amino-4,5-dialkoxy-4,5-dihydroimidazolines from 2-aminoimidazoles. J. Org. Chem..

[35] Huigens R.W., Ma L., Gambino C., Moeller P.D.R., Basso A., Cavanagh J., Wozniak D.J., Melander C. (2008). Control of bacterial biofilms with marine alkaloids derivatives. Mol. Biosyst..

[36] Zhang Y., Jiao N., Cottrell M.T., Kirchman D.L. (2006). Contribution of major bacterial groups to bacterial biomass production along a salinity gradient in the South China Sea. Aquat. Microb. Ecol..

[37] Delattre D., Pierre G., Laroche C., Michaud P. (2016). Production, extraction and characterization of microalgal and cyanobacterial exopolysaccharide. Biotechnol. Adv..

[38] Sullivan T., Regan F. (2017). Marine diatom settlement on microtextured materials in static field trials. J. Mater. Sci..

[39] Doiron K., Linossier I., Faÿ F., Yong J., Wahid E.A., Hadjiev D., Bourgougnon N. (2012). Dynamic approaches of mixed species biofilm formation using modern terchnologies. Mar. Environ. Res..

[40] Yang C., Song G., Zhu Q., Liu S., Xia C. (2016). The influence of bacterial quorum-sensing inhibitors against the formation of the diatom-biofilm. Chem. Ecol..

[41] Yang C., Fang S., Chen D., Wang J., Liu F., Xia C. (2016). The possible role of bacterial signal molecules N-acyl homoserine lactones in the formation of diatom-biofilm (*Cylindrotheca* sp.). Mar. Pollut. Bull..

[42] Alles M., Rosenhahn A. (2015). Microfluidic detachment assay to probe the adhesion strength of diatoms. Biofouling.

[43] McLachlan D.H., Underwood G.J.C. (2012). Calcium release form intracellular stores is necessary for the photophobic response in the benthic diatom navicular perminuta (bacillariophyceae). J. Phycol..

[44] Landoulsi J., Cooksey K.E., Dupres V. (2011). Interactions between diatoms and stainless steel: Focus on biofouling and biocorrosion. Biofouling.

[45] D’Costa P., Chandrashekar A. (2011). The effect of bacteria on diatom community structure—The “antibiotics” approach. Res. Microbiol..

[46] Hawkins M.L., Faÿ F., Réhel K., Linossier I., Grunlan I. (2014). Bacteria and diatom of silicones modified with PEO-silane amphiphiles. Biofouling.

[47] Faÿ F., Hawkins M.L., Réhel K., Grunlan M.A., Linossier I. (2016). Non toxic, anti-fouling silicones with variable PEO-silane amphiphile content. Green Mater..

[48] Faÿ F., Carteau D., Linossier I., Vallée-Réhel K. (2011). Evaluation of anti-microfouling activity of marine paints by microscopical techniques. Prog. Org. Coat..

[49] Kamjunke N., Spohn U., Füting M., Wagner G., Scharf E.M., Sandrock S., Zippel B. (2012). Use of confocal laser scanning microscopy for biofilm investigation on paints under field conditions. Int. Biodeterior. Biodegrad..

[50] Molino P.J., Campbell E., Wetherbee R. (2009). Development of the initial diatom microfouling layer on antifouling and fouling release surfaces in temperate and tropical Australia. Biofouling.

[51] Molino P.J., Childs S., Hubbard M.R.E., Carey J.M., Burgman M.A., Wetherbee R. (2009). Development of the primary bacterial microfouling layer on antifouling and fouling release coatings in temperate and tropical environments in Eastern Australia. Biofouling.

[52] Chen L., Xia C., Qian P.Y. (2017). Optimization of antifouling coatings incorporating butenolide, a potent antifouling agent via field and laboratory tests. Prog. Org. Coat..

[53] Stupak M.E., Garcia M.T., Pérez M.C. (2003). Non-toxic alternative compounds for marine antifouling paints. Int. Biodeterior. Biodegrad..

[54] Acevedo M.S., Puentes C., Carreno K., Leon J.G., Stupak M., Garcia M., Pérez M., Blustein G. (2013). Antifouling paints based on marine natural products from Colombian Caribbean. Int. Biodeterior. Biodegrad..

[55] Jellali R., Campistron I., Pasetto P., Laguerre A., Gohier F., Hellio C., Pilard J.F., Mouget J.L. (2013). Antifouling activity of novel polyisoprene-based coatings made from photocurable natural rubber derived oligomers. Prog. Org. Coat..

[56] Forjan E., Navarro F., Cuaresma M., Vaquero I., Ruiz-Dominguez M.C., Gojkovic Z., Vasquez M., Marquez M., Mogedas B., Bermejo E. (2015). Microalgae: Fast-growth sustainable green factories. Crit. Rev. Environ. Sci. Technol..

[57] Hellio C., Trepos R., Aguila-Ramirez R.N., Hernandez-Guerrero J., Stengel D.B., Connan S. (2015). Protocol for assessing antifouling activities of macroalgal extracts. Natural Products from Marine Algae.

[58] Thabard M., Gros O., Hellio C., Marechal J.P. (2011). Sargassium polyceratium (phaephyceae, fucaceae) surface molecule activity towards fouling organisms and embryonic development of benthic species. Bot. Mar..

[59] Tolker-Nielson T., Sternberg C., Filloux A., Ramos J.L. (2014). Methods for studying biofilm formation: Flow cells and confocal laser scanning microscopy. Pseudomonas Methods and Protocols.

[60] Heydorn A., Nielson A.T., Hentzer M., Sternberg C., Givskov M., Ersboll B.K., Molin S. (2000). Quantification of biofilm structures by the novel computer program comstat. Microbiology.

